# Transvaginal repair of enterocele following robot‐assisted radical cystectomy using a mesh for abdominal wall hernia repair

**DOI:** 10.1002/iju5.12497

**Published:** 2022-07-05

**Authors:** Tomoko Kuwata, Masami Takeyama, Masaki Watanabe, Hiromi Kashihara, Chikako Kato

**Affiliations:** ^1^ Head of Urogynecology Center Daiichi Towakai Hospital, The Department of Urology Takatsuki City Osaka Japan

**Keywords:** pelvic organ prolapse, radical cystectomy, transvaginal mesh

## Abstract

**Introduction:**

This report aims to describe our experience in the pelvic floor reconstruction of anterior enterocele following radical cystectomy by transvaginal surgery using a mesh for abdominal wall hernia repair.

**Case presentation:**

An 84‐years‐old woman developed pelvic organ prolapse 4 months after undergoing robot‐assisted radical cystectomy. After examination, she was diagnosed with a midline anterior enterocele. Considering the thinness of the vaginal wall and the large defect of the vaginal wall muscle layer, we performed transvaginal repair using a mesh for abdominal wall hernia repair designed to reduce the adhesion to the intestinal tract.

**Conclusion:**

At the 1‐year follow‐up, neither recurrence nor complications were observed. This showed that transvaginal mesh surgery for abdominal wall hernia repair could be a treatment option for pelvic organ prolapse with a vaginal wall muscle layer defect after radical cystectomy.

**Keynote message**

POP after RC is often difficult to repair because, in many cases, there may be little residual tissue for reconstruction. In those cases, pelvic floor reconstruction becomes possible by performing TVM surgery for abdominal wall hernia repair designed to reduce adhesion to the intestinal tract, and it could become one of the ideal treatment options.

**Abbreviations & Acronyms**
POP: pelvic organ prolapse
POP‐Q: pelvic organ prolapse quantification
RC: radical cystectomy
TVM: transvaginal mesh

## Introduction

POP following RC is rare, and surgical repair is more difficult due to the loss of pelvic floor tissues such, as connective tissue, muscles, and constituent tissues of the vaginal wall, caused by RC. In the literature, there are some reports on repair strategies: including transvaginal, laparoscopic, and transabdominal surgeries.^1^, ^2^, ^3^ For cases with many tissue defects, it may be necessary to use a mesh.^1^ This report aims to describe our experience in pelvic reconstruction using TVM.

### Case presentation

An 84‐year‐old woman was diagnosed with G3 (pT>2) bladder cancer and underwent robot‐assisted RC and ileal conduit diversion. She developed POP 4 months after the operation and tried a ring pessary, but it did not improve her symptoms. Eleven months after RC, she was referred to our hospital for POP repair.

**Fig. 1:**
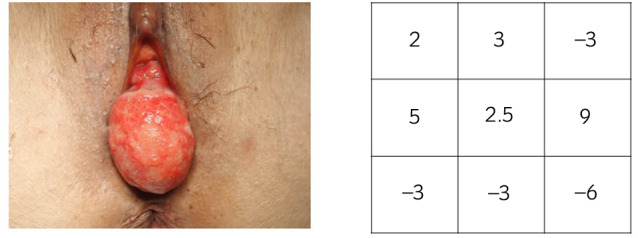
The patient presented with a midline enterocele with atrophic ulcerated vaginal skin. POP‐Q is shown.

**Fig. 2:**
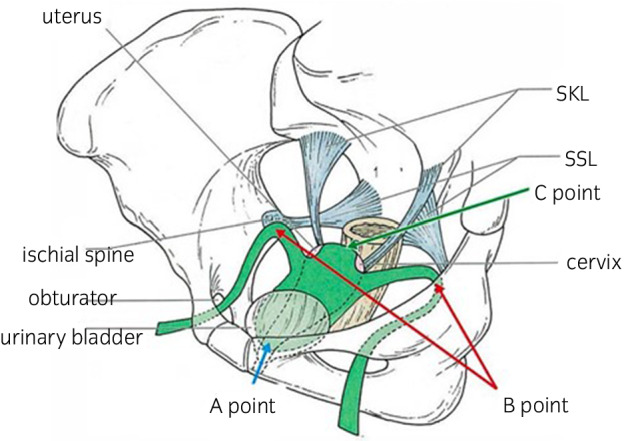
Anchoring points. Anchoring points are (1) the distal part of the anterior vaginal wall (A point), (2) the uterine cervix (C point), and (3) the firm tissue around both ischial spines (B point).

On the examination, she presented with a midline enterocele descending from the anterior vaginal side prior to the uterus with atrophic ulcerated vaginal skin (Fig. 1). Considering the thinness of the vaginal wall and the large defect of the vaginal wall muscle layer, we chose TVM surgery. Figure 2 shows a schematic diagram of the anterior wall TVM using mesh for pelvic floor repair. There are four anchoring points on the mesh: strong tissue around the bilateral ischial spines, the midline of the distal vaginal wall, and cervical tissue.

**Fig. 3:**
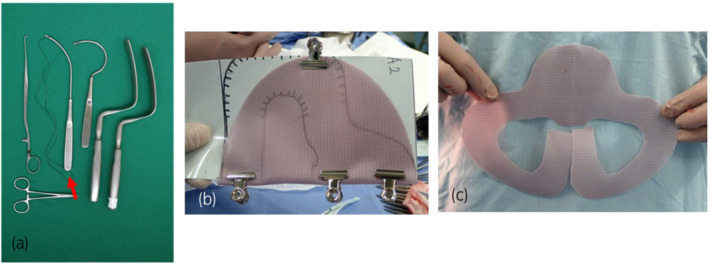
Instruments. The red arrow shows a weakly curved needle (a). Self‐cut mesh: we cut the shape shown on the right out of the mesh shown on the left based on a TVM pattern (b, c).

The patient underwent TVM surgery 13 months after RC. We made a vertical incision in the center of the vaginal wall with a scalpel and peeled off the vaginal wall sharply from the midline to the side. However, adhesion between the peritoneum and vaginal wall was so severe that the omentum was exposed. We changed the dissection layer laterally at the turned edge of the vaginal wall and proceeded to dissect sharply and bluntly toward the ischial spine. We made a small skin incision approximately 5 mm in length bilaterally, 5.5 cm laterally, and 2 cm dorsally from the clitoris base and penetrated the firm tissue (e.g., tendinous structures of muscles originating from the ischial spine) around the ischial spine using a weakly curved needle (Fig. 3). Then, we used nylon loops to guide the mesh arm.

After delamination, we used a non‐absorbable thread for mesh fixation over the midline of the distal vaginal wall and the cervix. As the repair should be performed within the abdominal cavity, we decided to use a monofilament polypropylene mesh (Ventralight™ ST; Bard Inc., Murray Hill, NJ, USA), which is used for abdominal wall hernia repair and designed to reduce adhesion to the intestinal tract on the ventral side (Fig. 3).

**Fig. 4:**
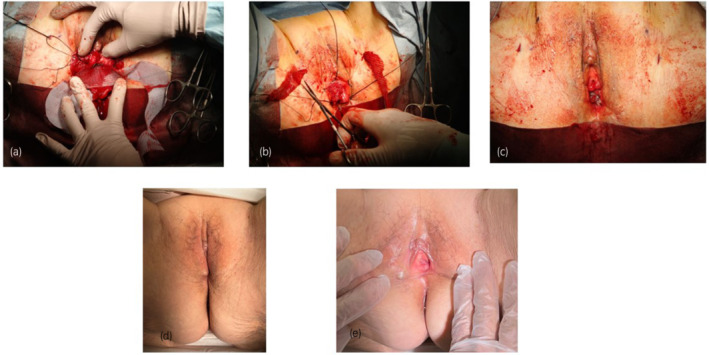
The mesh was fixed with a non‐absorbent thread, and the mesh arm was guided outside through the skin with a nylon loop (a, b). The condition after repair (c). One year after surgery (d, e).

And the mesh arm was guided outside through the skin with a nylon loop. Then the mesh was placed tension‐free. Finally we excised only the erosion site and performed a continuous suture to the vaginal wall with 3–0 Monokryl (Fig. 4). The operation time was 1 h and 45 min, and the amount of bleeding was 45 g.

At 1‐year follow‐up, neither recurrence nor complications, such as atrophic ulcerated vaginal skin related to the mesh and chronic pain were observed.

## Discussion

Since RC is a surgery to remove the bladder and pelvic tissues, such as the vaginal wall, uterus, and ovaries, post‐RC POP is among the potential postoperative complications and may cause serious morbidity. Although the incidence of postoperative POP among female patients who underwent RC is rare and the data on surgical management are insufficient,^4^ one study showed that the incidence of POP was 11% among patients who underwent simple cystectomy for interstitial cystitis.^5^ In POP following RC, it is more difficult to reconstruct the pelvic floor than normal POP repair because of the loss of tissue used for surgical repair. Although it is said that the success rate of colpocleisis is high in transvaginal surgery,^6^ it is necessary to use a mesh depending on the case.^1^, ^7^ Even though the use of mesh for transvaginal surgery is controversial, it is a useful option for achieving better results in the treatment of refractory POP with fragile tissues.^8^

In the present case, the vaginal wall was thin and the muscular layer defect was large, we selected TVN surgery. Intraoperative findings showed that separating the peritoneum from the vaginal wall was difficult. Therefore, we considered it inappropriate to use a conventional polypropylene mesh, such as Polyform™, which was still available for transvaginal surgery, due to the possibility of adhesion caused by long‐term contact with the intestinal tract. Thus, we attempted using a special monofilament polypropylene mesh (Ventralight™ ST). This mesh is originally used for abdominal wall hernia repair. It was designed to reduce adhesion to the intestinal tract, incorporating an absorbent hydrogel barrier based on Sepra® technology located on the ventral side. Besides the above‐mentioned characteristics, this mesh has the property that the coated part turns into a gel when exposed to a saline solution and the cut out mesh can be extended smoothly like the conventional mesh. Therefore, we were able to perform TVM surgery without any problems. There are various types of hernia meshes.^8^ It is necessary to select the appropriate mesh type depending on the approach of the surgery selected for pelvic floor reconstruction.^3^ When using TVM for POP with a peritoneum cavity, this mesh will be one of the options with good potential for pelvic floor reconstruction in terms of strength and usability.

There are various surgical reconstruction methods for post‐RC POP; thus, it is important to select the appropriate surgical method according to the case.

## Conclusion

The enterocele after RC was safely reconstructed with TVM surgery for abdominal wall hernia repair. One year after the operation, no recurrence, mesh exposure, or complications, such as ileus, were observed. This method suggests new possibilities for TVM.

## Author contributions

Masami Takeyama: Conceptualization; investigation; methodology. Masaki Watanabe: Validation. Hiromi Kashihara: Validation. Chikako Kato: Validation.

## Conflict of interest

The authors declare no conflict of interest.

## Approval of the research protocol by an Institutional Reviewer Board

N/A.

## Informed consent

N/A.

## Registry and the Registration No. of the study/trial

N/A.
